# Naringin and Naringenin in Liver Health: A Review of Molecular and Epigenetic Mechanisms and Emerging Therapeutic Strategies

**DOI:** 10.3390/antiox14080979

**Published:** 2025-08-10

**Authors:** Roberto Flores-Peña, Hugo Christian Monroy-Ramirez, Fernando Caloca-Camarena, Scarlet Arceo-Orozco, Jorge Alejandro Salto-Sevilla, Marina Galicia-Moreno, Juan Armendariz-Borunda

**Affiliations:** 1 Programa de Doctorado en Farmacología, Departamento de Fisiología, Centro Universitario de Ciencias de la Salud, Universidad de Guadalajara, Guadalajara 44340, Jalisco, Mexico; roberto.flores9900@alumnos.udg.mx (R.F.-P.); fernando.caloca@alumnos.udg.mx (F.C.-C.); 2 Instituto de Biologia Molecular en Medicina y Terapia Génica, Centro Universitario de Ciencias de la Salud, Universidad de Guadalajara, Guadalajara 44340, Jalisco, Mexico; hugo.monroyram@academicos.udg.mx (H.C.M.-R.); jorge.salto4084@alumnos.udg.mx (J.A.S.-S.); 3 Tecnologico de Monterrey, EMCS, Zapopan 45201, Jalisco, Mexico

**Keywords:** naringin, naringenin, flavonoids, polyphenols, liver disease, epigenetics modifications, natural products, nanocarriers, nanoparticles

## Abstract

Liver diseases have become a significant global health concern, driving increased interest in naturally occurring compounds as adjunctive treatments for managing these conditions. Flavonoids, a class of natural polyphenols found in plants and fruits, offer a broad spectrum of potential health benefits due to their applications in nutraceuticals, pharmaceuticals, and cosmetics. Among them, naringin (NARI) and naringenin (NAR), which are abundant in citrus fruits, have attracted considerable attention. Numerous studies have investigated the therapeutic effects of NARI and NAR across different stages of liver disease. This review highlights recent research on the impact of these flavonoids in alcohol-related liver disease and metabolic dysfunction-associated fatty liver disease (MASLD). It also explores the molecular mechanisms and epigenetic modifications through which NARI and NAR contribute to preventing liver disease progression. Finally, this work discusses recent progresses and future perspectives, emphasizing the role of innovative nanocarriers, nanoparticles, and drug delivery strategies in enhancing the efficacy and bioavailability of these promising compounds to improve liver health.

## 1. Introduction

Since ancient times, natural products have been used as remedies for various human diseases. Specifically, we refer to those compounds that are secondary metabolites produced by living organisms such as plants, animals, or microorganisms, which are recognized for their unique chemical composition and medicinal properties [1]. About a third of drugs approved by the Food and Drug Administration (FDA) in recent decades are based on an active ingredient obtained from natural products or their derivatives [2], with plant constituents being one of the primary sources of new drugs.

For several years, various research groups have investigated the effectiveness of polyphenols in promoting human health. Polyphenols are a group of naturally occurring compounds found in high concentrations in fruits, vegetables, spices, teas, and even red wine. Based on their chemical structure, they can be broadly classified into two major groups: flavonoids (including isoflavones, quercetins, cyanidins, and catechins) and phenolic acids (such as caffeic and ferulic acids) [3].

**Figure 1:**
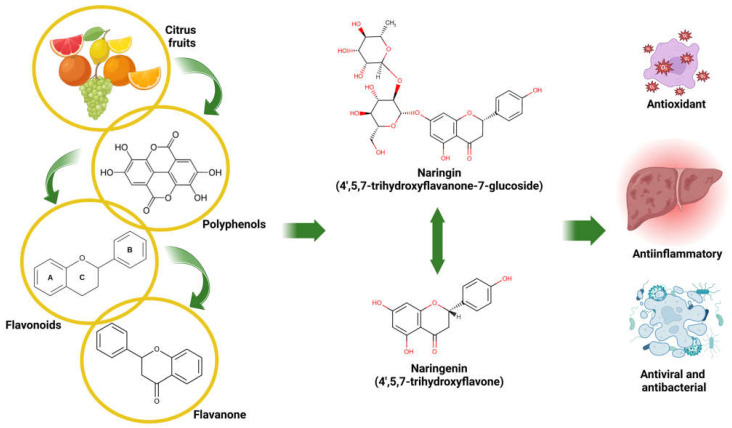
Origin, chemical structure, and medicinal properties of NARI and NAR. Created in BioRender. Galicia moreno, M. (2025) https://BioRender.com/m92v841.

Among the bioactive compounds present in citrus fruits, NARI and its aglycone form, NAR, stand out for their high concentration in fruits such as lemons, oranges, mandarins, and grapefruits (Figure 1). Both are flavanones that have been extensively studied in experimental models, both in vitro and in vivo, as well as in some clinical trials. They have been shown to possess a wide range of biological effects, including their antioxidant, anti-inflammatory, antitumor, antimicrobial (including antiviral, antifungal, and antibacterial activity), antiadipogenic, and cardioprotective properties. Of all these mechanisms, the most widely characterized is their antioxidant activity, which has prompted their study as therapeutic agents in various diseases [4,5]. In recent years, there has been interest in studying the potential of these flavonoids as gene and epigenetic modulators [6], which would expand their therapeutic potential in treating various diseases that affect humans.

On the other hand, liver disease is one of the leading causes of death worldwide, with more than two million deaths annually, many of them among people of working age, representing approximately 4% of global mortality. Leading causes include chronic alcohol consumption, responsible for 5.9% of deaths worldwide; hepatitis B and C virus infections, which cause approximately 1.3 million deaths each year; and, most notably, metabolic dysfunction-associated steatotic liver disease (MASLD), with approximately 3.3 million new cases annually and an estimated 280,000 deaths only in 2019 [7]. These conditions can progress to advanced stages such as cirrhosis or liver cancer, further exacerbating the burden of morbidity and mortality [8,9]. Data from both in vitro and in vivo reports indicate that these flavonoids regulate energy balance and enhance lipid and carbohydrate metabolism, as well as oxidative processes and inflammation, making them a promising therapeutic option for treating these diseases [10,11].

This work aims to comprehensively describe NARI and NAR’s chemical and pharmacological properties in treating pathologies affecting the liver gland. Novel aspects are addressed, including their capacity to regulate key genetic and epigenetic processes in these diseases, as well as their potential use as nanodrugs, and a description of new administration strategies.

## 2. Methods

PubMed and Google Scholar databases were searched using the following keywords: naringin; naringenin; flavonoids; polyphenols; liver disease; epigenetic modifications; natural products; nanocarriers; nanoparticles. There was no time limit, and the words “AND” and “OR” were used as logical combining terms, enabling us to limit the information. Original and review articles resulting from the search were selected following the main aim of this study. Furthermore, this study excluded unpublished articles and grey literature such as conference papers, theses, and patents. All the data shown in this work are included within the article.

## 3. NARI and NAR: Two Phytochemicals with Promising Health Benefits

### 3.1. Physicochemical and Pharmacological Properties of NARI and NAR

Phenolic compounds are bioactive agents found in over 10,000 species, most of which are part of the human diet, including fruits, vegetables, cereals, nuts, and processed foods such as tea, coffee, chocolate, and red wine. Moreover, polyphenols vary in the number of phenolic rings and hydroxyl groups they contain and can be broadly classified into flavonoids and non-flavonoids. The latter includes phenolic acids, stilbenes, and lignans [12,13,14].

On the other hand, flavonoids are natural compounds derived from plants that play key biological roles, such as contributing to the color and aroma of flowers and attracting pollinators in fruits. Additionally, they aid in plant growth, defend against ultraviolet radiation, and protect against pathogen attacks. These molecules offer various health benefits, making them valuable in nutraceuticals, pharmaceuticals, and cosmetics. This is due to their anti-inflammatory, antibacterial, antioxidant, antimutagenic, anti-aging, and anticancer properties. Furthermore, flavonoids can inhibit various enzymes, including xanthine oxidase (XO), cyclooxygenase (COX), lipoxygenase, and phosphoinositide 3-kinase [15,16]. These properties arise from the basic structure of fifteen carbon atoms arranged in a C6-C3-C6 configuration, forming two aromatic benzene rings, ‘A’ and ‘B’, and a heterocyclic pyran ring, ‘C’. Flavonoid compounds are typically categorized into six main subclasses: flavones, flavonols, flavanones, isoflavones, flavanols, and anthocyanins. The classification depends on the type of heterocycle to which the ‘B’ ring is attached, as well as the degree of oxidation or unsaturation of the ‘C’ ring [17].

Flavanones are found in high concentrations in citrus fruits, typically in their glycosylated form. They are also known as flavan-3-ols or dihydroflavones due to the saturation of the double bond between the 2- and 3-positions of the ’C’ ring. The primary aglycones of this subclass include NARI and NAR in grapefruit, eriodictyol in lemon, and hesperetin in orange [15].

Table 1 summarizes the physicochemical and pharmacological properties of NARI and its aglycone form, NAR.

### 3.2. NARI: Biotransformation and Metabolism

NARI has limited oral bioavailability, mainly due to its poor oral absorption, first-pass metabolism, and the elimination of its metabolites. In the intestine, phase II enzymes transform NARI into hydrophilic compounds, which are subsequently excreted from cells into the intestinal lumen by efflux transporter proteins. The free form of NARI has been transiently detected in the plasma of rats and humans; however, its presence is limited. Therefore, NAR glucuronide is considered the primary and most relevant metabolite for estimating serum NARI concentrations. This is because the intestinal microflora converts this glucuronide into its aglycone, NAR, facilitating its absorption [27,28]. This process occurs through the hydrolysis of NARI by the enzymes naringinase and L-rhamnosidase, resulting in the generation of prunin and rhamnose. Subsequently, prunin is degraded by the enzyme β-D-glucosidase to form NAR, which is then hydrolyzed by naringinase and rapidly absorbed in the gastrointestinal tract.

Alternatively, NARI can enter the liver via enterohepatic circulation, where it is conjugated to glucuronidated and sulfated metabolites, which can then be distributed to highly perfused organs such as the kidney, heart, and brain [5,29]. NARI is significantly less potent due to steric hindrance induced by sugar moiety, and it contributes to the bitter taste found in citrus juice and peel [30].

Finally, NARI is a bioactive compound with a favorable safety profile and low toxicity, and it is considered a relatively safe compound according to Organisation for Economic Co-operation and Development (OECD) criteria [31].

### 3.3. NAR: Biotransformation and Metabolism

NAR has limited oral absorption due to its low water solubility and extensive first-pass metabolism. It is absorbed in the intestine via both passive diffusion and active transport mechanisms [21,32]. After absorption, NAR is conjugated primarily in the gut and liver to form glucuronide metabolites (98% of NAR or β-d-glucuronide being detected as a metabolite absorbed) and sulfate metabolites in plasma. These conjugates circulate in the bloodstream non-covalently bound to serum albumin, facilitating their transport and distribution to organs with high blood perfusion, such as the liver, kidneys, heart, and brain [5]. Then, most NAR-conjugated metabolites, including NAR 7-glucuronide, NAR 7-sulfate 4′-glucuronide, and NAR 7-glucuronide 4′-sulfate, are mainly excreted via the biliary route, allowing their reabsorption into the enterohepatic circulation and increasing their half-life. On the other hand, NAR 4′-glucuronide, NAR 7-glucuronide, and NAR 7,4′-disulfate are excreted in urine, accounting for 7 to 23% of total metabolite excretion [21].

### 3.4. Key Pharmacokinetic Studies in Humans

Two pharmacokinetic studies have characterized NAR in humans, reporting variations likely related to differences in dosage, formulation, and analytical methods. Kanaze et al. evaluated 135 mg of pure NAR in six healthy volunteers, finding a Cmax of 2009.5 ± 770.8 ng/mL, a Tmax of 3.67 ± 0.82 h, a T½ of 2.31 ± 0.40 h, and an AUC (0−∞) of 9424.5 ± 2960.5 ng·h/mL. Urinary excretion accounted for 5.81 ± 0.81% of the administered dose [33].

In contrast, Rebello et al. conducted a dose-escalation study (150–900 mg) using sweet orange extract in 18 subjects, reporting a Cmax of 15.76 ± 7.88 μM and 48.45 ± 7.88 μM for the 150 mg and 600 mg doses, respectively, with similar Tmax and half-life values. Despite differences in formulation and measurement units (ng/mL vs. μM), both studies confirm that NAR exhibits low oral bioavailability and undergoes rapid metabolism. Importantly, no adverse events were associated with any dose, supporting the compound’s safety profile [34].

Both NARI and NAR are compounds with high antioxidant capacity; however, NARI is less potent than NAR. This difference is due to the steric hindrance caused by the presence of its sugar group, which partially blocks access to the active hydroxyl groups, thus limiting its effectiveness in neutralizing free radicals [23].

## 4. NARI and NAR in Liver Diseases: Efficacy, Safety, and Insights into Gene Regulatory Mechanisms

### 4.1. Alcohol-Related Liver Disease (ALD)

Liver disease caused by excessive alcohol consumption (112−210 g/per week for 5 years or more) is one of the leading causes of hepatic steatosis and, consequently, chronic liver damage. It is estimated that approximately three million people die from excessive alcohol consumption [35]. This disease encompasses a series of disorders ranging from fatty liver to alcoholic hepatitis and eventually might end in cirrhosis and/or hepatocarcinoma.

In general, once alcohol enters the body, it is primarily absorbed in 75% in the intestinal tract and only 1% is eliminated in its original form through feces. Once it crosses the vascular endothelium, the remaining percentage reaches the bloodstream. It is metabolized in the liver by oxidation reactions to acetaldehyde, thanks to the enzymatic activity of alcohol dehydrogenase, microsomal oxidases, and catalase. Along with obtaining these metabolites, this biotransformation process favors the production of Reactive Oxygen Species (ROS) and an imbalance in the NAD/NADH ratio [36]. Then, acetaldehyde must be metabolized again, a limiting process regulated by acetaldehyde dehydrogenase, finally generating acetic acid. Another alternative is that the acetaldehyde formed is conjugated with Coenzyme A (CoA) through the participation of acetyl-CoA synthetase, generating acetyl-CoA, the key to starting the tricarboxylic acid cycle.

Ethanol harms the liver through oxidative stress, endoplasmic reticulum stress, inflammation, and apoptosis. It also deregulates lipid metabolism, allowing lipids to accumulate in the liver parenchyma. This subsequently favors the development of steatohepatitis or, in more severe cases, hepatocarcinoma [37].

Regarding the pharmacological management of alcoholic liver disease (ALD), prednisolone remains the standard treatment for alcoholic hepatitis; however, its primary limitation is that it improves only short-term survival. Consequently, various research groups have focused on identifying new pharmacological targets, including exploration of the gut–liver axis, the use of anti-inflammatory agents, antioxidants, and molecules capable of promoting liver regeneration, all of which may represent promising therapeutic strategies. The role of flavonoids in restoring liver function has been extensively studied, with numerous preclinical and clinical studies demonstrating their efficacy and safety [38].

In a liver damage model, using zebrafish larvae incubated in a 2% ethanol solution for 32 h and subsequently treated with 6.25, 12.5, and 25 mg/L of NARI for 48 h, it was observed that treatment with this flavonoid decreased steatosis and liver injury induced by ethanol exposure. Its antioxidant and anti-apoptotic effects were observed in this damage model. There is evidence of the impact of NAR on the metabolism of fatty acids and cholesterol, specifically diminishing the activity of fatty acid synthase (FASN), glucose-6-phosphate dehydrogenase (G6PD), and hepatic beta-oxidation. FASN plays a fundamental role in lipogenesis by catalyzing the synthesis of long-chain saturated fatty acids from acetyl-CoA, malonyl-CoA, and nicotinamide adenine dinucleotide phosphate (NADPH). At the same time, G6PD is a lipogenic enzyme involved in supplying NADPH for the biosynthesis of fatty acids. Therefore, decreased enzymatic activity of FASN and G6PD in the liver may limit the availability of long-chain fatty acids required for triglyceride synthesis [39].

In 2010, Jayaraman, J. et al. observed the hepatoprotective effect of NAR in an experimental ethanol-induced liver damage model. In a murine model, rats administered a 20% ethanol solution daily for 60 days, while daily administration of NAR (50 mg/kg) was performed during the last 30 days of the experiment. In this trial, NAR administration had positive impacts by decreasing the levels of bilirubin, alkaline phosphatase, substances reactive to thiobarbituric acid (TBARS), and conjugated dienes and increasing the activity of the enzymes alcohol dehydrogenases (ADH), acetaldehyde dehydrogenase (ALDH), superoxide dismutase (SOD), and catalase (CAT). This in vivo trial shed light on the potential effectiveness of NAR in preventing alcohol-induced liver damage [40].

For their part, Zhao, L. et al. compared the efficacy of various flavonoids (apigenin, quercetin, genistein, epigallocatechin, and NAR in mice in which ALD was induced by intragastric administration of ethanol (50%) for 5 weeks. In this study, genistein and NAR were the only treatments effective in mitigating the fibrogenic response and the apoptotic process activated by the damage caused by alcohol [41].

Although the hepatoprotective effect of NAR has been little explored both preclinically and clinically, some studies have analyzed the benefit of this molecule at other levels. Soliman, N.A. et al., in 2023, demonstrated that the administration of 50 mg/kg/day of NAR had a positive effect on neurodegeneration caused by chronic ethanol consumption in rats, improving cognitive dysfunction and neuroprotection by acting on signaling pathways such as those mediated by nuclear factor erythroid-related 2 (Nrf2), and its target molecules such as NADPH quinone oxidoreductase 1, heme oxygenase-1 (HO-1), improving antioxidant capacity; in addition, it increased the levels of ciliary neurotrophic factor, favoring neuroprotection, in addition to attenuating necroptosis. These findings suggest that NAR can treat alcohol-induced neurotoxicity [42].

Recently, a model of ethanol damage evaluated the efficacy of Scenedesmus-derived extracellular vesicles loaded with NAR. This release system, combined with NAR, allowed an increase in the dose of this flavonoid from 0.2 to 1.0 mg/mL, attenuated the increase in liver damage markers: aspartate aminotransferase (AST), alanine aminotransferase (ALT), alkaline phosphatase (ALP), and triglycerides (TG). Also, it improved oxidative damage and liver inflammation. Activation of the nuclear factor Nrf2/HO-1 and regulation of the expression of CYP2E1 were observed; a modification in the intestinal microbiota composition was also observed. Pharmacologically, this formulation allows a sustained release of the active ingredient and high cytocompatibility. In summary, this new formulation improves the efficacy of NAR during the alcohol-induced liver damage experimentally [43].

There is a lack of studies that verify the effects that NAR exerts at a systemic level on the inflammatory process and the changes that occur in the brain–gut-liver axis; in addition, its safety must be evaluated in randomized-placebo controlled clinical studies considering the different types of consumption and the degree of liver damage that patients with this disease may have. Expanding these findings to consider these molecules as therapeutic options for treating this disease is necessary.

### 4.2. Non-Alcoholic Liver Disease: Viral Hepatitis

Viral hepatitis is defined as inflammation of the liver caused by infection with one of five biologically unrelated viruses. These viruses are the primary cause of the global burden of disease. The five viruses are hepatitis A (HAV), B (HBV), C (HCV), D (HDV), and E (HEV). These agents are known as hepatotropic viruses [44,45]. Each of these viruses possesses distinctive characteristics in terms of biological structure, transmission mechanisms, host adaptation, disease progression, epidemiologic distribution, and response to antiviral treatment [46]. Viral hepatitis can be acute or chronic. Prolonged inflammation of the liver can lead to fibrosis, irreversible cirrhosis, and eventually HCC [47]. While in some cases, acute hepatitis may spontaneously resolve, in other instances, it can progress to a state of chronic infection [44].

Viral hepatitis constitutes a significant global cause of liver-related morbidity and mortality, affecting millions of individuals and imposing a substantial economic burden despite the availability of effective treatments [45,48]. In response to this pressing public health concern, the World Health Organization (WHO) has designated July 28th as World Hepatitis Day and initiated a comprehensive global strategy to enhance this condition’s detection, treatment, education, and vaccination. By 2030, the WHO has set ambitious targets to reduce hepatitis-related mortality by 65%, decrease new infections by 90%, and ensure that at least 80% of the population has access to essential treatment services [48,49].

Flavonoids have been reported to exhibit antiviral activity, as they act on viral replication by inhibiting viral adhesion and entry into host cells, inhibiting transcription, and intervening in different stages of viral DNA replication. Also, they influence protein translation and the assembly, packaging, and release of new viral particles. Flavonoids have also been shown to modulate the immune system, helping to reduce viral load [50,51].

A recent study suggests that NAR may inhibit hepatic steatosis induced by HBV X protein. This protein integrates into the host’s DNA during hepatocellular regeneration following each hepatitis outbreak. NAR’s mechanism of action involves the suppression of the transcriptional activity of SREBP1c, liver X receptor α (LXRα), and peroxisome proliferator-activated receptor γ (PPARγ) factors in both HBx-expressing transgenic mice and HBx-transfected HepG2 cells [52].

It has been demonstrated that targeting the host metabolic pathways on which the virus depends could deprive the virus of critical resources needed to complete its life cycle. In this regard, Goldwasser, J. et al. demonstrated that NAR acts as a non-toxic inhibitor by blocking the assembly of very low-density lipoprotein (LDL) and HCV infectious particles in Huh7.5.1 cells and primary human hepatocytes [53]. Furthermore, Nahmias, Y. et al. strengthened this evidence by demonstrating that NAR, at concentrations below the toxic threshold, reduced HCV secretion by up to 80% in primary human hepatocytes and in infected mouse models [54].

While the evidence for using NARI or NAR in viral hepatitis remains limited, studies with various flavonoids have demonstrated the potential for these compounds to interfere with key stages of the viral life cycle. This mechanism of action suggests that flavonoids may have great potential as an adjunctive therapy in treating viral hepatitis. However, further preclinical and clinical studies are necessary to validate their efficacy and safety in humans and to establish their therapeutic use with scientific support.

### 4.3. Non-Alcoholic Liver Disease: Metabolic Dysfunction-Associated Fatty Liver Disease

MASLD is the most current term for a group of liver diseases characterized by an abnormal deposition of fat in hepatocytes [55]. NAFLD, as it was previously named, was diagnosed when other liver diseases, such as viral hepatitis and excessive alcohol consumption, were absent. Therefore, the diagnosis was considered exclusive. Currently, the term MASLD allows diagnosis for patients with concomitant liver diseases while excluding those without any metabolic risk factors, including type 2 diabetes mellitus (T2DM), hypertension, obesity, and dyslipidemia. According to the latest European Association for the Study of the Liver (EASL) and American Association for the Study of Liver Diseases (AASLD) guidelines, in the presence of hepatic steatosis and at least one of cardiometabolic risk factors confer a diagnosis of MASLD [56], which include parameters such as body mass index (BMI) ≥ 25 kg/m^2^ (≥23 kg/m^2^ in people of Asian ethnicity); waist circumference: ≥94 cm in men and ≥80 cm in women (Europeans), ≥90 cm in men and ≥80 cm in women (South Asians and Chinese), ≥85 cm in men and ≥90 cm in women (Japanese); blood pressure ≥ 130/85 mmHg or treatment for hypertension; plasma TG ≥ 1.7 mmol/L or lipid-lowering treatment; plasma high-density lipoprotein cholesterol (HDL) ≤ 1.0 mmol/L in men and ≤1.3 mmol/L in women or lipid-lowering treatment; fasting plasma glucose (levels 100–125 mg/dL or 2 h post-load); glucose levels (140–199 mg/dL) or glycosylated hemoglobin (HbA1c) between 5.7 and 6.4% [57].

Currently, MASLD affects up to 30% of adults worldwide, with its prevalence increasing from 22% to 37% between 1991 and 2019. This global trend closely mirrors the obesity crisis, impacting up to 70% of individuals who are overweight and more than 90% of those with morbid obesity [58,59]. A recent systematic review identified the highest prevalence of MASLD in the Latin American population at 44.37%, followed by the Middle East and North Africa region (36.53%), South Asia (33.83%), Southeast Asia (33.07%), North America (31.20%), East Asia (29.71%), the Asia-Pacific region (28.02%), and Western Europe (25.10%). The same study, published in 2023, reported that individuals with MASLD have an annual mortality rate of 12.6 per 1,000 when diagnosed using ultrasound and/or the Fatty Liver Index (FLI). This rate increased to 17.05 per 1,000 when biopsy was included as a diagnostic method [60]. The reported prevalence of MASLD in Mexico in 2006 was about 14%. However, by 2018, this had an increment that went to 49.9%, highlighting its strong correlation with obesity [61]. Metabolic dysfunction-associated steatohepatitis (MASH) represents the most severe spectrum of MASLD. Histologically, it is characterized by lobular inflammation and hepatocyte ballooning. This condition is associated with a higher risk of advanced liver fibrosis, cirrhosis, and HCC [58].

Currently, treatments for MASLD are limited and scarce; the most used approach focuses on reducing steatosis, mitigating liver damage, and addressing the metabolic consequences of the disease while lowering cardiovascular risk. The primary recommendation is caloric restriction through dietary modifications to achieve a weight loss of 7–10%. In some cases, pharmacological interventions may enhance weight loss more effectively [62,63]. Proper nutrition and daily and controlled exercise remain the most accepted strategies for managing MASLD.

In recent years, compounds such as NAR have garnered significant interest due to highlights on their therapeutic potential in MASLD. For example, Yu, R. et al. [64] demonstrated that administering mice a diet containing 0.1% NAR for 16 weeks slowed weight gain and reduced TG, serum total cholesterol (TC), and liver TC levels. Additionally, it decreased the infiltration of dendritic cells (DCs) and macrophages in colon tissues and inhibited the production of inflammatory factors in both mice intestinal and liver tissues. Moreover, NAR improved intestinal permeability by enhancing the expression of tight junction proteins and reversed intestinal dysbiosis. These findings align with the results reported by Mu, H. et al. [65].

Another study in mice revealed that administering NAR at a dose of 100 mg/kg/day for one week attenuated hepatic lipid accumulation and inflammation in the livers of mice on a methionine and choline-deficient diet. The effects were pronounced in wild-type (WT) mice, while NLRP3 −/− mice exhibited less hepatic steatosis than WT mice; however, NAR did not further reduce hepatic steatosis in the NLRP3 −/− group. The same researchers showed that NAR inhibited activation of the NLRP3/NF-κB pathway induced by oleic acid (OA) and lipopolysaccharides in Kupffer cells. It also reduced lipid deposition and the expression of NLRP3 and IL-1β in WT hepatocytes [66]. Experimental models of MASLD in rats have also shown promising results. A study published in 2021 reported that oral administration of NAR at doses of 10, 30, and 90 mg/kg for two weeks significantly reduced serum levels of TG and TC, while improving liver function by decreasing the transaminases AST and ALT. The same study also demonstrated that NAR treatment in cell lines reduced lipid accumulation in a dose-dependent manner and promoted AMPK pathway activation, thereby enhancing mitochondrial biogenesis, regulating autophagy, and increasing energy expenditure, mechanisms that could contribute to the treatment of MASLD and other metabolic disorders [67].

Additionally, NAR has been investigated in clinical settings. The first clinical trial, a double-blind, randomized, placebo-controlled study, evaluated the effects of NAR in patients with MASLD. Participants received 100 mg of NAR twice daily for four weeks. The intervention significantly reduced the severity of the disease, as well as serum levels of TG, TC, and LDL. It also increased HDL levels compared to the placebo group [68].

In conclusion, NARI and NAR have shown promising therapeutic potential in preclinical studies in the context of MASLD, attributable to their antioxidant, anti-inflammatory, and lipid metabolism-modulating properties. Their main advantages include their natural origin, low toxicity profile, and ability to target multiple pathophysiological pathways. However, the lack of further clinical evidence limits their therapeutic application. Therefore, well-designed clinical studies are required to validate their efficacy and safety in humans, thus advancing their potential incorporation as adjuvant agents in the treatment of MASLD.

### 4.4. Drug-Induced Liver Injury (DILI)

The liver plays a central role in drug metabolism and detoxification, rendering it particularly susceptible to damage associated with this process [69]. DILI was first described by Popper and Schaffner [70] as an injury to the liver or biliary system resulting from the metabolism of a drug. This condition can cause morphological changes in liver cells, oxidative stress, inflammation, apoptosis, necrosis, mitochondrial dysfunction, and organ dysfunction. This health problem represents the leading cause of acute liver failure in the Western world [68,71].

Increased use of herbal products and dietary supplements, combined with limited regulatory oversight in their manufacturing, has raised significant public health concerns globally, particularly in Western countries [72,73].

Susceptibility to hepatotoxicity is influenced by factors such as age, sex, body weight, genetics, race, ethnicity, pre-existing liver disease, comorbidities, and obesity. External factors such as alcohol consumption, smoking, diet, and drug characteristics (physicochemical properties and dosage) also increased risk [68,74,75,76]. Women have a 1.5 to 1.7-fold higher risk of adverse drug reactions than men, a risk that doubles after the age of 50, possibly due to menopause-related changes [77].

The diagnosis of DILI requires establishing a link between drug exposure and liver injury while excluding other potential causes. However, identifying cases of DILI remains challenging, limiting its understanding compared to other liver diseases [72]. Globally, determining the true incidence of DILI is difficult due to cultural, traditional, and healthcare system differences, as well as inconsistent reporting and definitions [67,78]. Epidemiologic studies suggest that antibiotics, anticonvulsants, and psychotropic drugs are the leading causes of DILI in Europe and the United States. At the same time, herbal remedies and dietary supplements are the leading causes in Asia [67].

Epidemiologic data suggest that antibiotics in Western countries and anti-tuberculosis drugs in Asian countries are the major causes of idiosyncratic DILI worldwide. In the United Kingdom and the United States, acetaminophen (APAP) is the most common cause of DILI, accounting for approximately 50% of cases of acute liver failure. In Asia, on the other hand, cases associated with traditional Chinese medicine and dietary supplements are more prevalent [75]. Mexico has the second-highest registered medicinal plant species (4,000), which is exceeded only by China (5,000). However, despite efforts to identify the different herbal remedies used in Mexico, data on herbal consumption and DILI injuries are largely lacking [79].

This condition compromises drug safety, poses a significant health risk, and drives up healthcare costs [80]. Understanding and studying DILI is critical, but its investigation is hampered by the lack of reliable laboratory tests to identify the causative drug [74].

Noteworthy, natural products are emerging as potential treatments for DILI. Sun Y.K. et al. found that NARI reduced APAP-induced hepatic steatosis and inflammation by decreasing TNF-α and lipid peroxidation while increasing IL-4, reduced glutathione (GSH), and antioxidant enzymes such as glutathione peroxidase (GPx), SOD and CAT [81]. Also, Khaled, S.S. et al. evaluated the effect of NARI and/or NAR as a treatment against hepatic toxicity induced by Taxol. The authors demonstrated that administering these compounds reduced serum levels of total bilirubin, lactate dehydrogenase (LDH), AST, ALT, ALP, and γ-glutamyl transpeptidase (GGT) in Taxol-treated rats. Finally, they suggested that the mixture of NARI and NAR was the most potent in improving liver function and structural integrity [82]. Also, NAR has demonstrated potent hepatoprotective effects against dasatinib-induced liver toxicity. Dasatinib, a second-generation tyrosine kinase inhibitor used in the treatment of acute lymphoblastic leukemia and chronic myeloid leukemia, is associated with hepatotoxicity through the increased production of proinflammatory mediators (TNF-α, IL-10, IL-6) and a decrease in antioxidant enzymes (CAT, SOD, GPx, GST). NAR effectively lowers ALT and AST levels, reduces inflammation, and restores antioxidant defenses, highlighting its critical role in preventing dasatinib-induced liver injury [83].

Tuberculosis treatments such as isoniazid (INH) and rifampicin (RIF) are major contributors to DILI due to the generation of ROS that induce lipid peroxidation [84]. Wang, C. et al. demonstrated that pretreatment with NAR reduced INH- and RIF-induced increases in ALT, AST, and malondialdehyde (MDA) levels, highlighting its protective role through inhibition of oxidative stress [85]. Similarly, doxorubicin, an antineoplastic drug for solid tumors, is associated with hepatotoxicity. Wali, A.F. et al. reported that NAR effectively attenuated doxorubicin-induced liver injury by reducing oxidative stress and inflammation, which are major contributors to its toxicity [86].

In conclusion, both NARI and NAR have demonstrated hepatoprotective potential in the context of DILI, a condition that is one of the leading causes of acute liver failure. The increasing use of herbal products and dietary supplements, coupled with inadequate regulatory oversight in their manufacturing, has raised significant concerns about health risks, especially in Western countries. Despite advances in DILI research, diagnosis remains difficult due to the lack of reliable tests to identify the causative agent. In this regard, these flavonoids have shown promising effects in experimental models, including the treatment of hepatotoxicity induced by APAP, Taxol, dasatinib, and INH, among others. Since oxidative stress is one of the main mechanisms that allow the development of this disease, the antioxidant and anti-inflammatory capacities exerted by both molecules are relevant to counteract it.

The findings above position NARI and NAR as potential therapeutic options, not only to mitigate the toxic effects of drugs, but also to improve the structural and functional integrity of the liver affected by drug use.

### 4.5. Hepatocellular Carcinoma (HCC)

**Figure 2:**
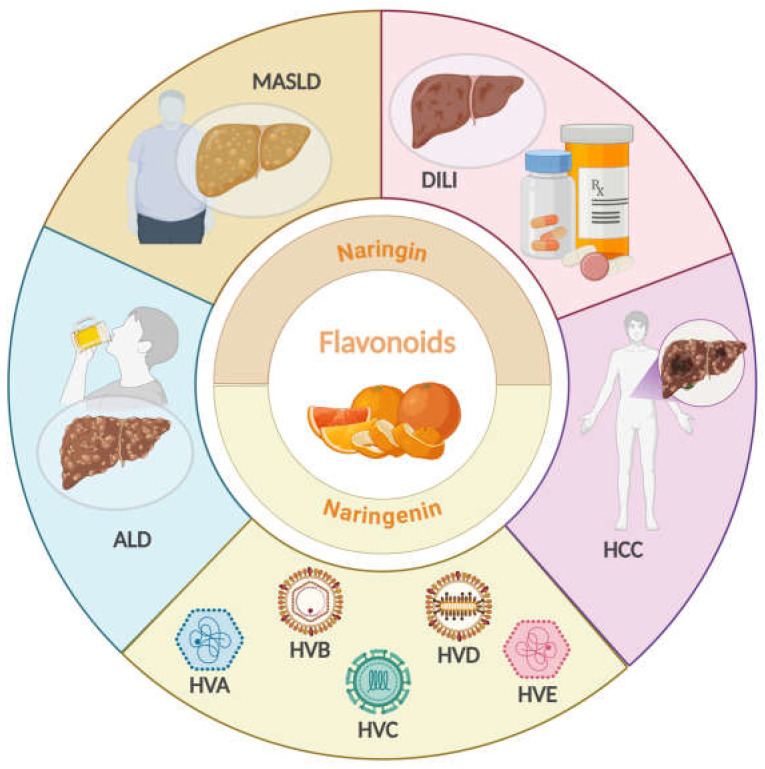
Natural compounds, NARI and NAR as therapeutic agents for liver disease. Created in BioRender. Galicia moreno, M. (2025) https://BioRender.com/h35a792.

HCC is the most common form of primary liver cancer and represents a challenge for healthcare systems worldwide due to its high mortality rate [87]. Its epidemiology is linked to the presence of chronic liver diseases, such as those caused by HBV and HCV, excessive alcohol consumption, and metabolic disorders, such as MASLD (Figure 2) [88].

Due to the complexity of its physio pathogenesis, the number of treatment options is limited, and the effectiveness of available treatments is often limited such as liver transplantation, local ablative therapies or surgical resection, which are more effective if performed in the early stages of the disease [89]. Due to the above, it is necessary to evaluate the efficacy of molecules that have been effective in treating other liver diseases and determine where these and their different mechanisms of action can be useful for the pharmacological management of HCC.

Cancer is a complex pathological process. Various molecular damage pathways are active, such as the pro-oxidant and pro-inflammatory response, which disrupt normal proliferation and the cell cycle, favoring tumor formation [90].

Much has been said about the health benefits of consuming citrus and berries, mainly due to their high flavonoid content. Therefore, administering NARI and NAR can help treat this disease positively. Banjerdpongchai, R. et al. observed that NARI was effective in inducing apoptosis of HepG2 cells through the activation of caspase-9, -8, and -3, favoring the expression of the proapoptotic protein Bcl-2 and decreasing the mitochondrial transmembrane potential [91]. For their part, Xie, D. et al. evaluated the proapoptotic effect of NARI by expressing miR-19b in the HepG2 cell line. They observed that NARI was effective in inhibiting the proliferation of HepG2 cells, and morphological analyses suggested that this flavonone induces cell contraction and chromatin condensation; in addition, stimulation with NARI increased the expression of miR-19b, which participates in the apoptosis of tumor cells [92].

In addition, NARI effects have been evaluated using nanotechnology. Wang, W. et al. assessed the impact of NARI-loaded nanoparticles in an aflatoxin-induced HCC model in rats. A dose of 6.18 mg/kg b.w. was effective in counteracting the oxidative damage and inflammation generated, presumably due to its positive effect on its bioavailability [93]. Moreover, Mohamed, E.E. et al. evaluated the efficacy of NARI and dextrin nanoparticles loaded with NARI (10 mg/kg b.w.) in HCC induced by the co-administration of DEN and 2-AAF. Both treatments showed beneficial effects on tumorigenesis, oxidative stress, and the inflammatory process, in addition to promoting the apoptosis process and preventing hyperproliferation of liver cells [94]. Finally, in a recent study carried out by Elwan, A.G. et al. the efficacy of nanoparticles with NAR was evaluated in an in vitro model using HepG2 and WI38 cells. The study concluded that this nanoformulation is effective in inhibiting cell proliferation and inducing apoptosis in HepG2 cells. More importantly, nanoparticles with NAR did not show cytotoxic effects in normal cells [95].

In summary, the pharmacological management of HCC continues to represent a clinical challenge. Currently available therapies are often difficult to access due to their high costs, and their safety and efficacy profiles remain limited. In this context, the evaluation of natural compounds emerges as a promising strategy to expand the available therapeutic options. In preclinical models of HCC, both NARI and NAR have demonstrated, in addition to their recognized antioxidant and anti-inflammatory effects, the ability to induce apoptosis and inhibit specific cell proliferation. While further research is still required to validate these findings, the current results suggest emerging therapeutic potential for these molecules in the treatment of HCC.

## 5. NARI and NAR as Epigenetic Modulators and Their Impact on Treating Liver Diseases

### 5.1. Epigenetic Targets in Liver Diseases Landscape

**Figure 3:**
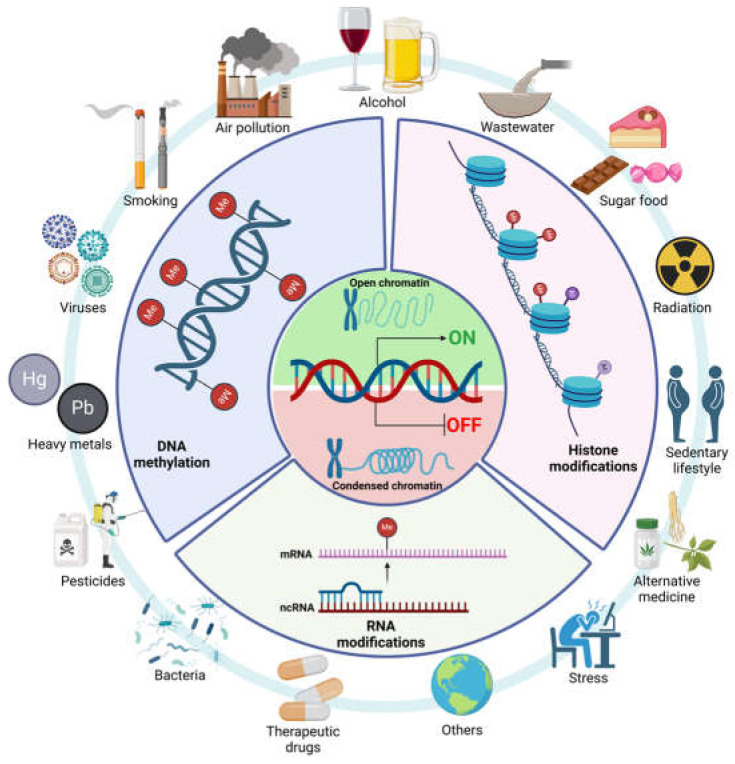
Epigenetic modifications and risk factors influencing gene expression and silencing. Created in BioRender. Galicia moreno, M. (2025) https://BioRender.com/i57l001.

Epigenetics refers to changes in gene expression that do not result from variations in the underlying DNA sequence. Various factors, including environmental factors, lifestyle, and dietary components such as flavonoids, can influence these alterations [96]. Epigenetic alterations may encompass DNA methylation, the process of adding a methyl group to DNA, typically resulting in reduced gene expression (hypermethylation), histone modification, or chemical alterations in histone proteins, which enclose DNA, influencing its accessibility for transcription and non-coding RNAs (ncRNAs) affecting messenger RNAs (mRNA), thereby altering gene expression (Figure 3) [97].

Although all cells in a multicellular organism share a typical genome, each presents a specific phenotype. As previously described, epigenetics refers to a set of processes that can influence gene expression and cellular phenotype in response to interaction with the environment interaction. The study of epigenetics can provide a better understanding of chronic diseases and improve diagnostic and therapeutic tools in any branch of medicine. Furthermore, since epigenetic modifications are highly plastic and respond to stimuli, epigenetic therapy is of great interest, enabling both the development of new drugs and the use of natural alternatives [98].

The group of chronic liver diseases includes HCC, MASLD, and advanced-liver fibrosis represents a worldwide health problem, directly or indirectly topping morbidity and mortality lists, with significant costs to health systems throughout the world. And it seems that a compelling question in the field of hepatology needs to be how only a small portion of patients with chronic hepatic disorders develop severe symptoms or complications, while the vast majority remain asymptomatic or slowly evolving throughout their lives. In addition to the genetic landscape of background mutations, different changes in gene expression directly related to the pathogenesis and progression of liver disease have been linked to the rearrangement of the epigenomic landscape [99].

Several studies have demonstrated a strong association between aberrant DNA methylation patterns and the presence of specific single-nucleotide polymorphisms (SNPs) that influence the severity of metabolic dysfunction-associated fatty liver disease (MAFLD). Among these, the rs738409 C > G SNP in the PNPLA3 gene, which results in the I148M (Ile148Met) amino acid substitution, has been extensively characterized as a genetic modifier of MAFLD. This variant has been associated with increased hepatic fat accumulation, enhanced susceptibility to fibrosis, and a higher risk of progression to HCC. Aberrant DNA methylation can alter gene expression and interact with the effects of PNPLA3 variants, further amplifying the severity of liver damage [100,101].

On the other hand, hepatic control of lipid and carbohydrate metabolism is modified by histone acetylase enzymes. Regular circadian changes in lipid synthesis are associated with dynamic histone acetylation patterns of target genes in liver chromatin. This is controlled by histone deacetylase 3 (HDAC3), and its depletion promotes the development of hepatic steatosis. Moreover, HDAC3 influences transcriptional repression of lipogenic genes, and its absence results in increased lipid accumulation due to dysregulated expression of enzymes involved in fatty acid synthesis. Additionally, HDAC3 interacts with nuclear receptors and corepressor complexes, further modulating metabolic gene expression. These findings suggest that HDAC3 plays a critical role in maintaining hepatic metabolic homeostasis and preventing lipid overload in the liver.

One of the best-studied deregulated master chromatin remodelers in HCC is enhancer of zeste homolog 2 (EZH2), the catalytic subunit of the polycomb repressive complex 2. EZH2 is overexpressed in HCC and is associated with malignant progression, vascular invasion, and cell proliferation. In vivo studies in nude mice have identified EZH2 as a crucial regulator of HCC tumorigenesis. Intratumoral EZH2 knockdown resulted in significant tumor regression, supporting its potential as a therapeutic target [102].

### 5.2. Natural Compounds with Epigenetic Targets and Their Use in Liver Diseases

Natural compounds with epigenetic targets have gained interest in their therapeutic potential in liver diseases. Epigenetic modifications such as DNA methylation or demethylation, histone acetylation or deacetylation, and microRNA regulation play critical roles in liver pathophysiology by influencing gene expression without altering the DNA sequence. Various natural compounds have demonstrated epigenetic modulatory effects with promising results in hepatic pathologies. Curcumin, a polyphenol derived from turmeric, exhibits inhibitory properties against histone acetyltransferase (HAT) and histone deacetylase (HDAC), thereby reactivating tumor suppressor genes and inhibiting the proliferation of liver cancer cells [103]. Epigallocatechin-3-gallate (EGCG), a catechin found in green tea, has been shown to inhibit DNA methyltransferase (DNMT), contributing to the re-expression of silenced genes involved in cell cycle regulation and apoptosis in HCC models [104].

Resveratrol, a polyphenol in grapes, exerts HDAC inhibition and microRNA modulation. Studies suggest that it may reduce liver fibrosis and improve NAFLD by regulating lipid metabolism and inflammation [105]. Additionally, sulforaphane, a compound derived from cruciferous vegetables, has been shown to modulate both DNMT and HDAC activity, improving liver detoxification and reducing oxidative stress [106].

These natural compounds offer a dual advantage in liver diseases: they correct aberrant epigenetic modifications and exert anti-inflammatory, antioxidant, and antiproliferative effects. Their use may aid in restoring normal gene expression patterns, reducing hepatic inflammation, and preventing malignant transformation. However, further randomized, placebo-controlled clinical studies are required to validate their efficacy, optimal dosing, and long-term safety in patients with liver diseases.

### 5.3. Epigenetic Regulation Modulated by NARI and NAR, an Opportunity Area for the Therapeutic Positioning of These Flavonoids: Basic and Clinical Evidence

Several studies have suggested that NARI and NAR may influence these epigenetic mechanisms in various ways. Research indicates that NARI may affect DNA methylation, a process that modulates gene expression, resulting in the suppression of gene expression. Alterations in DNA methylation are associated with numerous diseases, including cancer and metabolic disorders. NARI has been observed to cause significant changes in DNA methylation patterns in colon cancer, suggesting a possible mechanism for exerting anticancer therapeutic properties [6].

Conversely, Madureira, M.B. et al. suggest that a combination of NAR and hesperidin may regulate the epigenetic mechanisms of the Wnt/β-catenin signaling pathway, along with PI3K/AKT, p53, and MAPK, which are implicated in cellular growth, proliferation, survival, differentiation, migration, metabolism, and apoptosis [107]. Moreover, this combination had an inhibitory effect on histone acetylation. Wang, S.W. et al. demonstrated that these flavonoids synergistically protected pancreatic β-cells both in vivo and in vitro, and this action was independent of their intrinsic antioxidant properties [108].

Regarding the regulation of NARI through Non-coding RNA, Fan, J. et al. demonstrated that this molecule increased miR-20a expression levels, leading to a decrease in PPARγ protein expression in bone marrow stem cells within osteoblasts. The findings indicate that miR-20a may regulate PPARγ expression in BMSCs. This is the first study to report NARI-induced osteogenesis via the upregulation of miR-20a expression levels [109]. The anticancer effect of NARI is demonstrated by its ability to suppress small cell lung cancer cells growth through the miR-126/vascular cell adhesion molecule 1 (VCAM-1) signaling pathway [110]. Furthermore, Xie, D. et al. demonstrated that NARI could induce apoptosis in HepG2 cells and increase the expression of miR-19b mRNA. In addition, it can potentially elevate Bax protein expression and reduce Bcl-2 protein expression during apoptosis [92].

Fan, W. et al. conducted a study using messenger RNA sequencing (mRNA-seq), microRNA sequencing (miRNA-seq), and real-time quantitative PCR to identify expression differences between control and NAR-treated HepaRG cells. A total of 1,037 mRNAs and 234 miRNAs exhibiting differential expression were identified. Subsequently, twenty potential miRNA-mRNA pairs associated with liver metabolism were identified through target prediction and in silico integration analysis. Notable biochemical pathways include the PI3K-Akt signaling, cytokine-cytokine receptor interaction, viral carcinogenesis, EMC receptor, focal adhesion, neuroactive ligand-receptor interaction, phagosome, and changes linked to various cancers, including gastric cancer, prostate cancer, and human papillomavirus infection. These findings provide new insights into how NAR may affect the regulation of miRNAs and mRNAs that are related to metabolism [111]. On the other hand, Lozano-Herrera S.J. et al. analyzed how in vitro co-exposure of a fermented NAR extract with bisphenol A affects HT-2 colon cancer cells. This study demonstrated that NAR enhances the activation of intrinsic cell death pathways through phosphatase and tension homolog (PTEN), while increasing the activity and expression of caspase 9 through miR-200c and miR-141. These findings are relevant to understanding the molecular effects exerted by these compounds [112].

Clinical evidence supports the use of these modifiable epigenetic marks as potential therapeutic targets in HCC. While several of these drugs have demonstrated efficacy, either individually or in combination, their clinical use remains controversial, primarily due to safety concerns [113]. Despite the above, there is currently no clinical evidence exploring the effect of NARI or NAR in treating liver diseases. However, their ability to modulate epigenetic marks has been postulated as a mechanism of action. Therefore, well-designed clinical studies are needed to substantiate this hypothesis.

In summary, several studies indicate that NARI and NAR are capable of modulating complex epigenetic markers. However, both molecules still need to overcome numerous challenges before reaching human clinical trials that confirm the results observed in experimental models. Although accumulating evidence has enriched our understanding of their diverse molecular mechanisms, conflicting data persist, hampering their clinical translation. Furthermore, uncertainty about the duration of their effect on epigenetic regulation and their pharmacokinetic limitations prevents them from being considered as first-line treatments.

Regardless of that, the ability of NARI and NAR to modulate epigenetics constitutes a promising area of research. The use of integrative omics approaches, and the development of improved pharmaceutical formulations could provide additional robust evidence to establish their safety and efficacy. Rigorously designed clinical trials are needed to support these opportunities. In the long term, these flavonoids could be considered as epigenetic modulators in personalized medicine strategies for various liver diseases.

Table 2 provides a summary of the studies reviewed in the previous sections regarding the mechanisms of action of NARI and NAR.

## 6. Nanoformulations and Emerging Strategies to Optimize the Delivery of NARI and NAR

**Figure 4:**
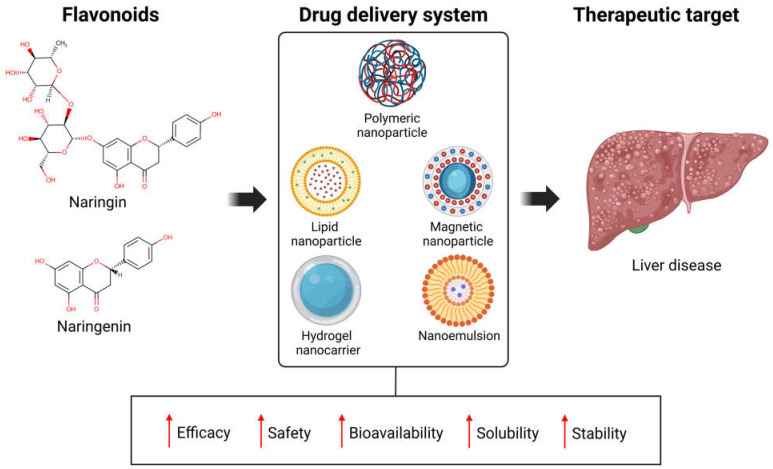
NARI and NAR formulations such as nanodrugs, through incorporation in different drug delivery systems, might result in improved outcomes in the treatment of liver diseases. Created in BioRender. Galicia moreno, M. (2025) https://BioRender.com/m28t149. ↑ increased.

The bioavailability of NARI and NAR is very low due to their hydrophobic nature, first-pass metabolism, and short half-life, which limit their therapeutic potential. These challenges have prompted efforts to enhance their pharmacokinetic and pharmacodynamic properties. Nanoscale drug delivery systems (with a size ≤ 100 nm) have emerged as a promising strategy in recent years. Nanoformulations offer several advantages, including protecting the drug during storage and administration and enabling controlled release and targeted delivery to specific organs like the liver. These systems enhance therapeutic performance by improving efficacy, safety, bioavailability, solubility, stability and reducing undesirable side effects (Figure 4) [114].

In the following lines, we will describe how nanotechnology improves the biological efficacy of both NAR and NARI.

### 6.1. Polymeric Nanoparticles

Polymeric biomaterials can be categorized into two main types: naturally derived and synthetic polymers. Natural polymers are derived from biological sources and offer several significant advantages, including biocompatibility and enzymatic degradation. These include polypeptides like collagen, gelatin, silk fibroin, polysaccharides such as sodium alginate, chitosan, cellulose, and hyaluronic acid derivatives, among others [115,116]. However, their use as drug carriers has been limited by challenges such as broad molecular weight distributions and batch-to-batch variability [117].

Unlike natural polymeric biomaterials, synthetic biomaterials offer enhanced processability and mechanical properties. They also exhibit excellent biodegradability, biocompatibility, and functional versatility [118,119]. These materials undergo degradation by breaking molecular chains in vivo, with the degradation metabolites being absorbed or excreted without causing secondary damage to human health [120]. Synthetic biomaterials encompass a range of products, including gels, nanofibers, and sponges, which can be composed of materials such as polyvinyl alcohol (PVA), polycaprolactone (PCL), polylactic acid (PLA), poly (lactic-co-glycolic acid) (PLGA), polyurethane (PU), and polyethylene oxide/polyethylene glycol (PEO/PEG) [121].

On the other hand, a murine study conducted by Fan, S. et al. developed a negatively charged PLGA-PEG nanoparticle system to encapsulate NARI for treating ulcerative colitis. The surface of these nanoparticles was further coated with chitosan and mannose to enhance their mucosal adsorption and macrophage-targeting capabilities. When administered orally (NARI dose of 10 mg/kg, b.w.), the nanoparticles demonstrated excellent resistance to degradation in the gastrointestinal environment, targeted the colon effectively, mitigated the symptoms of induced colitis, downregulated oxidative stress, and restored the gut microbiota [122].

NAR, present in the Si-Ni-San (a traditional Chinese formulation composed of four herbs including the root of *Bupleurum chinense* DC, the root of *Paeonia lactiflora* Pall, the fruit of *Citrus aurantium* L, and the root of *Glycyrrhiza uralensis* Fisch) has been reported to inhibit breast cancer growth and metastasis induced by chronic psychological stress. According to the authors, Si-Ni-San was administered to mice by oral gavage at concentrations of 0.825 g/kg (low dose) and 1.65 g/kg (high dose). Additionally, NAR was administered by oral gavage at doses of 10 and 20 mg/kg b.w. This effect is mediated through the modulation of estrogen metabolism via the FXR/EST pathway in the liver [123]. In the study by Zhao, Y. et al., a novel nano endocrine drug, NAR-cell penetrating peptide-galactose nanoparticles (NCGs), was reported. NCGs demonstrated specific hepatic targeting and enhanced intestinal barrier permeability in cell xenograft and zebrafish models. Moreover, after oral administration NCGs (at a dose of 6.9 mg/kg b.w. of NAR administered orally) showed hepatic targeting and enterohepatic circulation in mouse breast cancer xenografts. Notably, the cancer inhibitory efficacy of NCGs was superior to monomeric NAR and the positive control, tamoxifen. Additionally, NCGs treatment increased hepatic estrogen sulfotransferase expression and reduced estradiol levels in the liver, blood, and breast tumor tissue of female C57BL/6 mice. Importantly, NAR in NCGs exhibited excellent stability upon oral administration and did not cause side effects, such as coagulation disorders, endometrial thickening or osteoporosis [124].

Also, in the study by Elnawasany, S. et al. developed NAR-loaded PLGA nanoparticles using the precipitation method, which exhibited the smallest size, and the highest drug encapsulation efficiency (*p* < 0.05) compared to those prepared by the simple emulsion solvent evaporation technique. These nanoparticles effectively inhibited the proliferation of HepG2 cells in a dose-dependent manner, with IC_50_ values of 7.25 ± 0.17 μM at 24 h and 5.21 ± 0.18 μM at 48 h. Additionally, they increased the levels of p53, Bax, caspase-3, and cytochrome C, decreased the expression of Bcl-2 compared to the control, and enhanced cytotoxic activity [125].

### 6.2. Lipid Nanoparticles

Liposomes, nanoemulsions, solid lipid nanoparticles, and nanostructured lipid carriers are lipid-based nanocarriers. Liposomes are spherical lipid vesicles composed of one or more lipid bilayers with a hydrophilic core [126].

In the study by Pareek, A. et al., a novel dual-drug delivery system was developed to simultaneously deliver NAR and doxofylline (DOX) to the lungs for the treatment of asthma. This system included NAR-loaded glyceryl tristearate-based solid lipid nanoparticles (NAR-SLN), which were then further loaded with DOX into chitosan tripolyphosphate-based swellable microspheres (NAR-SLN-DOX-sMS). In vitro drug release studies demonstrated rapid release of DOX, with a cumulative drug release of 50.5% within the first two hours, and sustained release of NAR, with a 72.3% drug release over 12 h from NAR-SLN-DOX-sMS (at a dose of 10 mg of the lyophilized). Furthermore, in vivo studies (300 mg/kg b.w., dry powder via inhalation) revealed a significant reduction in BAL fluid eosinophils, serum bicarbonate levels, histamine release, and bronchial inflammation compared to asthmatic mice, with results closely resembling those of normal mice [127].

Bronchial hyperreactivity in mice treated with microspheres was significantly reduced compared to the asthmatic group. Histopathological evaluation further confirmed the efficacy of the optimized inhalable microspheres, as no damage to the bronchial wall was observed. Therefore, it can be concluded that this novel inhalable dual-drug delivery system for DOX and NAR represents a promising alternative for the effective treatment of asthma.

### 6.3. Magnetic Nanoparticles

Magnetic nanoparticles (MNPs) are a unique class of nanoparticles (often made from materials like iron, nickel, or cobalt) and have a size between 1 and 100 nanometers. One of the most unique properties of magnetic nanoparticles (MNPs) is superparamagnetism [128].

In the study by Askar, M.A. et al., dextran-coated magnetic nanoparticles loaded with curcumin and NAR (CUR-NAR-D-MNPs) were investigated as chemotherapy agents and in combination with radiotherapy (RT) to assess their efficacy in suppressing breast cancer both in vitro and in vivo. The nanoparticles were prepared through controlled chemical coprecipitation of the magnetite phase from aqueous solutions of Fe^2+^ and Fe^3+^. After treatment of MCF-7 cells with CUR-NAR-D-MNPs or RT for 48 h, a significant increase in ROS levels was observed compared to the control group. CUR-NAR-D-MNPs exhibited immune modulation and anti-inflammatory properties, suggesting that the combined curcumin-NAR formulation can convert cancer cells into non-cancerous cells by reducing the expression of CD44 and TNF-α. Therefore, this nanomaterial demonstrates good biocompatibility, with the ability to stimulate ROS production, induce tumor cell apoptosis, and inhibit tumor cell proliferation. Noteworthy, the authors reported the preparation of these nanoparticles by adding dextran-coated MNPs (20 g) to a mixture containing 2 g of curcumin and 2 g of NARI, which had been previously dissolved in 750 mL of ethanol [129].

### 6.4. Hydrogel Nanocarriers

Hydrogel is a porous three-dimensional network structure composed of highly hydrated polymer chains cross-linked by chemical or physical means [130].

In the study by Elkhoury, K. et al., bioactive nanocomposite hydrogels were developed, consisting of nano-sized liposomal building blocks made from salmon-derived lecithin, loaded with NARI (at concentrations of 25, 50, and 100 μg/mL), and macro-sized hydrogels of gelatin methacryloyl for embedding. This formulation takes advantage of the significant drug loading capacity of liposomes, their role in reinforcing the hydrogel network, and the injectability and light-mediated cross-linking of bioderived gelatin-based biomaterials. Administration of this NARI-loaded nanocarrier to human adipose tissue-derived stem cells confirmed its cytocompatibility. The results of this study provide evidence that these nanoliposomes are highly biocompatible and can be safely used for bone tissue engineering applications [131].

Recently, a three-dimensional structure based on a chitosan hydrogel incorporating a NARI/β-cyclodextrin inclusion nano complex (NARI/CD-HCh) has been designed and applied at a concentration of 2 mg/mL of NARI/β-cyclodextrin. This nano complex is biocompatible and effective in promoting wound contraction in an animal model. The results demonstrated that the fabricated hydrogels exhibited outstanding physical and biological properties, making them highly beneficial for wound healing and regenerative medicine applications [132].

On the other hand, Elshabrawy, H.A. et al. utilized 3D bioprinting and electrospinning technologies to develop a transdermal double-layer patch (TDDP) for the treatment of rheumatoid arthritis (RA). The first layer consists of a hydrogel made using 3D printing, incorporating hyaluronic acid and dexamethasone, while the second layer is composed of electrospun polycaprolactone nanofibers loaded with NARI at a concentration of 10 mg/mL. In vivo experiments confirmed the efficacy of the newly developed TDDP, demonstrating a significant reduction in the levels of proinflammatory cytokines, such as IL-6 and TNF-α, as measured by sandwich ELISA in plasma samples from *Rattus norvegicus* [133]. In a related study, a layered microneedle (MN) patch was developed to address challenges in burn healing. The MN patch features a core/shell structure, with the bottom layer consisting of methacrylated gelatin encapsulating hypoxia-induced exosomes derived from human umbilical vein endothelial cells, while the top layer contains NARI-loaded CaCO_3_ nanoparticles. Upon application of the MN patch to the thermal burn injury site, the microneedles puncture the eschar, leading to the spontaneous degradation of the NARI-loaded CaCO_3_ nanoparticles in the interstitial fluid. This degradation triggers the sustained release of NARI, which helps alleviate local inflammation and scavenge excess ROS. The MN patch successfully promoted scarless healing of skin burn injuries in rat models, providing a potential approach for the thermal treatment of burns in clinical settings [134].

### 6.5. Nanoemulsions

Oil-in-water (O/W) and water-in-oil (W/O) nanoemulsions are two types of nanoemulsions that are created when two immiscible liquids are dispersed and stabilized by adding the right surfactants and co-surfactants, with the correct particle size being approximately 100 nm [135].

In the study by Jun, H. et al., a preclinical trial is reported involving the intratracheal administration of NARI-loaded biomimetic nanoparticles coated with stem cell membranes (CM-NARI-NPs), which were prepared using the emulsification and evaporation method, for the treatment of acute lung injuries. The results demonstrated that, in vitro, CM-NARI-NPs exhibited good dispersibility, biocompatibility, and biosafety (up to 80 µg/mL of NARI did not induce significant cytotoxicity). At the cellular level, CM-NARI-NPs effectively targeted inflamed macrophages and exhibited strong ROS-scavenging capabilities. In vivo imaging showed that CM-NARI-NPs could target and accumulate in inflamed lungs. Moreover, intratracheal instillation of the nanoparticles (at a dose of 10 mg/kg, b.w. of NARI) significantly reduced ROS levels, inhibited proinflammatory cytokines, and notably improved the survival rate. Finally, CM-NARI-NPs increased the expression of the M2 marker (CD206) and decreased the expression of the M1 marker (F4/80) in septic mice, indicating that NARI-modulated macrophages were polarized toward the M2 subtype [136].

In summary, the development of nanoformulations represents a significant advance in the field of drug delivery, especially for compounds such as NARI and NAR, whose pharmacokinetic properties are limited by their low oral bioavailability. Through the design of nanoparticles, nanoemulsions, liposomes, and other systems, their pharmacokinetic profile has been improved, allowing for more efficient absorption and sustained release at the site of action.

However, despite these advantages, particular challenges associated with their application must be considered. These include the complexity of synthesizing and standardizing nanostructures, high production costs, and the lack of large-scale clinical studies to support their long-term safety. Furthermore, aspects such as the biocompatibility of the materials used, the potential for toxicity, and the environmental impact of nanomaterial waste still require thorough evaluation.

Finally, while nanoformulations present a promising path for boosting the therapeutic effects of natural compounds like NARI and NAR, their clinical use requires careful, balanced, and responsible evaluation that considers both their advantages and limitations. Ongoing development and refinement of these nanoformulations could lead to new and effective treatments for liver diseases, cancer, and various chronic inflammatory conditions.

## 7. Conclusions and Perspectives

Flavonoids are widely studied molecules due to their antioxidant properties, primarily attributed to the presence of phenolic chemical groups in their structure. Like many other antioxidants currently under development or already applied in clinical settings, their effect primarily lies in their free radical-scavenging activity and their ability to enhance the activity of antioxidant enzymes. Additionally, various studies have demonstrated that many of these molecules can modulate complex molecular mechanisms and important signaling pathways, thereby strengthening their beneficial action in treating multiple diseases, including liver diseases.

NARI and NAR are promising molecules for treating liver diseases due to their potent antioxidant, anti-inflammatory, and anti-apoptotic properties. These flavanones have been the focus of numerous preclinical studies that support their efficacy in liver protection by modulating different signaling pathways related to oxidative stress and inflammation, among others. The above have been postulated as adjuvant therapies capable of improving the condition of the damaged liver, preventing the progression of the disease to more severe stages such as cirrhosis or HCC.

Although there is sufficient basic evidence of the promising results of these molecules in experimental models of liver injury, clinical findings remain limited, which prevents the extrapolation of results obtained from basic studies to what occurs in patients with liver disease. The available clinical evidence has demonstrated the efficacy of these molecules in improving alterations in lipid profiles, reducing systemic inflammation, and positively modulating insulin resistance, all of which are key factors in the development of metabolic liver disease. Furthermore, considerable attention has been devoted to the impact of epigenetic regulation on the development of various diseases. As described above, NARI and NAR fulfill this function by modulating DNA methylation, histone modification, and regulating noncoding RNA, increasing their arsenal of pharmacodynamic mechanisms that help restore hepatic homeostasis.

On the other hand, the application of nanotechnology in releasing and administering these flavanones promises to improve their pharmacokinetic parameters, enhancing their efficacy and safety. In this sense, the data presented in this study provide a glimpse of a promising future for developing new nanoformulations that incorporate NARI and NAR as active ingredients in the treatment of various diseases.

Finally, the absence of clinical trials should be seen as an opportunity to evaluate the efficacy and safety of these molecules in the treatment of liver diseases. They are consumed as food supplements, but their mechanisms of action go beyond their nutritional function.

In conclusion, despite scientific advances, it is essential to continue interdisciplinary research to optimize their pharmacokinetic properties and clarify their pharmacodynamic mechanisms in humans within a broader healthcare context. Although preclinical data have been solid, well-controlled randomized clinical trials are essential to validate NARI and NAR’s therapeutic efficacy and safety rigorously.

## Figures and Tables

**Table 1:** Physicochemical and pharmacological properties of NARI and NAR.

	NARI	NAR	Reference
Name	4′,5,7-trihydroxyflavanone-7-glucoside	4′,5,7-trihydroxyflavone	[18,19]
Molecular Formula	C_27_H_32_O_14_	C_15_H_12_O_5_	
Molecular Weight (g/mol)	580.4	272.3	
Melting Point	83 °C	251 °C	
Oral Bioavailability	5 to 9%	15%	[20,21]
Solubility (µg/mL)	No data	43.83	[22]
LD50 (g/kg of BW)	>5 (in BD) >16 (in SDR)	>5 (in BD)	[23,24,25,26]
NOAEL (mg/kg of BW/per day)	500 (in BD) >1250 (in SDR)	No data	[23,24]

NARI, naringin; NAR, naringenin; LD50, median lethal dose; BD, beagle dogs; SDR, Sprague-Dawley rats; BW, body weight; NOAEL, no observed adverse effect level. Created in Microsoft^®^ Word for Microsoft 365 MSO (version 2505 build 16.0.18827.20102).

**Table 2:** Summary of the mechanisms of action of NARI and NAR.

Name of Flavonoid	Mechanism/Outcome	References
NARI	Promotes the metabolism of fatty acids and cholesterol by decreasing the enzymatic activity of FASN and G6PD in the liver, thereby limiting the availability of long-chain fatty acids required for triglyceride synthesis.	[39]
	Anti-inflammatory and lipid-lowering effects, with decreased ALT, AST, TG, and TC levels. NARI also reduced the expression of Srebp1, Fas, Acc, Scd1, TNF-α, and IL-6.	[65,82,93]
	Antioxidant activity, with increased SOD and GPx enzyme activity and reduced ROS levels.	[82,93]
	Proapoptotic effect through activation of CASP-8 and CASP-9, induction of Bid proteolysis, and miR-19b expression.	[91,92]
	Anticancer effect, reducing hepatic expression of Bcl-2, IQGAP1, IQGAP3, Ras signaling, Ki-67, and miR-126, while counteracting the decrease in VCAM-1. It also increased the expression of IQGAP2, Bax, p53, ATG5, LC3, PDCD5, and miR-126.	[92,94,95,110,111]
	Osteogenic effect, increasing miR-20a levels and reducing PPARγ expression.	[109]
NAR	Antioxidant activity, with increased ADH, ALDH, SOD, CAT, and GPx enzyme activity, inhibition of p300/CBP acetyltransferase, and reduced Txnip expression.	[40,81,82,85,86,108]
	Antifibrotic effect, reducing TGF-β, COL-I, and fibronectin expression, along with signs of fibrosis in the liver.	[41]
	Neuroprotective and antioxidant activity, with increased Nrf2, GSH, and NQO1 expression, and reduced RIPK3 expression.	[42,43]
	Modulates hepatic apolipoprotein and lipid synthesis, reducing ALT, AST, CHOL, and TG levels, and decreasing mRNA expression of PPARγ and LXRα.	[52]
	Antiviral effect through PPARα activation, reducing VLDL production and inhibiting ApoB secretion by decreasing MTP and ACAT activity and expression.	[53,54]
	Anti-inflammatory and lipid-lowering activity, with decreased TG and TC levels, and negative regulation of NLRP3, TNF-α, IL-1β, IL-6, IL-18, p65, MCP-1, COX-2, NF-κB, F4/80, and MPO activity, via AMPK pathway activation.	[64,66,67,68,81,82,83,86]
	Anticancer effect, increasing miR-200c and miR-141 expression.	[112]

NARI, Naringin; NAR, Naringenin; FASN, Fatty Acid Synthase; G6PD, Glucose-6-Phosphate Dehydrogenase; ALT, Alanine Aminotransferase; AST, Aspartate Aminotransferase; TG, Triglycerides; TC, Total Cholesterol; Srebp1, Sterol Regulatory Element-Binding Protein 1; Fas, Fatty Acid Synthase; Acc, Acetyl-CoA Carboxylase; Scd1, Stearoyl-CoA Desaturase 1; TNF-α, Tumor Necrosis Factor-alpha; IL, Interleukin; SOD, Superoxide Dismutase; GPx, Glutathione Peroxidase; ROS, Reactive Oxygen Species; CASP, Caspase; Bid, BH3 Interacting Domain Death Agonist; miR, MicroRNA; Bcl-2, B-cell lymphoma 2; IQGAP, IQ Motif Containing GTPase Activating Protein; Ras, type of G protein; Ki-67, Marker of Proliferation Ki-67; VCAM-1, Vascular Cell Adhesion Molecule 1; Bax, BCL-2-Associated X Protein; p53, Tumor Protein 53; ATG5, Autophagy-Related 5; LC3, Microtubule-associated Protein 1A/1B-light Chain 3; PDCD5, Programmed Cell Death 5; PPARγ, Peroxisome Proliferator-Activated Receptor Gamma; ADH, Alcohol Dehydrogenase; ALDH, Aldehyde Dehydrogenase; CAT, Catalase; p300/CBP, p300/CBP coactivator; TGF-β, Transforming Growth Factor Beta; COL-I, Collagen Type I; Nrf2, Nuclear Factor Erythroid 2-Related Factor 2; GSH, Glutathione; NQO1, NAD(P)H Quinone Dehydrogenase 1; RIPK3, Receptor-Interacting Serine/Threonine Kinase 3; CHOL, Cholesterol; mRNA, Messenger RNA; LXRα, Liver X Receptor Alpha; VLDL, Very Low-Density Lipoprotein; ApoB, Apolipoprotein B; MTP, Microsomal Triglyceride Transfer Protein; ACAT, Acyl-CoA Cholesterol Acyltransferase; NLRP3, NOD-Like Receptor Family Pyrin Domain Containing 3; p65, NF-κB p65 Subunit; MCP-1, Monocyte Chemoattractant Protein-1; COX-2, Cyclooxygenase-2; NF-κB, Nuclear Factor-kappa B; F4/80, Macrophage Marker Antigen; MPO, Myeloperoxidase; AMPK, AMP-Activated Protein Kinase. Created in Microsoft^®^ Word for Microsoft 365 MSO (version 2505 build 16.0.18827.20102).

## Abbreviations

The following abbreviations are used in this manuscript: AASLD (American Association for the Study of Liver Diseases), AD (Alzheimer’s disease), ADH (alcohol dehydrogenases), ALD (Alcoholic Liver Disease), ALDH (acetaldehyde dehydrogenase), ALP (alkaline phosphatase), ALT (alanine aminotransferase), APAP (acetaminophen), AST (aspartate aminotransferase), BD (beagle dogs), BMI (body mass index), BW (body weight), CAT (catalase), CM-NARI-NPs (naringin-loaded biomimetic nanoparticles coated with stem cell membranes), CoA (coenzyme A), COX (cyclooxygenase), CRP (C-reactive protein), CUR-NAR-D-MNPs (dextran-coated magnetic nanoparticles loaded with curcumin and naringenin), DCs (dendritic cells), DILI (drug-induced liver injury), DNA (deoxyribonucleic acid), DNMT (DNA methyltransferase), DOX (doxofylline), EASL (European Association for the Study of the Liver), EGCG (epigallocatechin-3-gallate), EZH2 (enhancer of zeste homolog 2), FASN (fatty acid synthase), FDA (Food and Drug Administration), FLI (fatty liver index), G6PD (glucose-6-phosphate dehydrogenase), GGT (gamma-glutamyl transferase), GPx (glutathione peroxidase), GSH (reduced glutathione), HAT (histone acetyltransferase), HAV (hepatitis A virus), HbA1c (glycosylated hemoglobin), HBV (hepatitis B virus), HCC (hepatocellular carcinoma), HCV (hepatitis C virus), HDAC (histone deacetylase), HDAC3 (histone deacetylase 3), HDL (high-density lipoprotein cholesterol), HDV (hepatitis D virus), HEV (hepatitis E virus), HO-1 (heme oxygenase-1), HOMA (homeostatic model assessment), INH (isoniazid), LD50 (median lethal dose), LDH (lactate dehydrogenase), LDL (low-density lipoprotein), LXRα (liver X receptor α), MAFLD (metabolic dysfunction-associated fatty liver disease), MASH (metabolic dysfunction-associated steatohepatitis), MASLD (metabolic steatotic disease associated with dysfunction), MDA (malondialdehyde), miRNA-seq (microRNA sequencing), MN (microneedle), MNPs (magnetic nanoparticles), mRNA (messenger ribonucleic acids), NADPH (nicotinamide adenine dinucleotide phosphate), NAFLD (non-alcoholic fatty liver disease), NAR (naringenin), NARI (naringin), NARI/CD-HCh (three-dimensional structure based on a chitosan hydrogel incorpo-rating a naringin/β-cyclodextrin inclusion nanocomplex), NAR-SLN (naringenin-loaded glyceryl tristearate-based solid lipid nanoparticles), NAR-SLN-DOX-sMS (naringenin-loaded glyceryl tristearate-based solid lipid nanoparticles loaded with doxofylline onto chitosan tripolyphosphate-based swellable microspheres), NCGs (naringenin-cell penetrating peptide-galactose nanoparticles), ncRNAs (non-coding ribonucleic acids), NOAEL (no observed adverse effect level), Nrf2 (nuclear factor erythroid 2-related factor 2), O/W (oil-in-water), OECD (Organisation for Economic Co-operation and Development), PCL (polycaprolactone), PEO/PEG (polyethylene oxide/polyethylene glycol), PLA (polylactic acid), PLGA (poly (lactic-co-glycolic acid)), PPARγ (peroxisome proliferator-activated receptor γ), PTEN (phosphatidylinositol-3,4,5-trisphosphate 3-phosphatase), PU (polyurethane), PVA (polyvinyl alcohol), RA (rheumatoid arthritis), RIF (rifampicin), ROS (reactive oxygen species), RT (radiotherapy), SDR (sprague-dawley rats), SOD (superoxide dismutase), T2DM (type 2 diabetes mellitus), TBARS (substances reactive to thiobarbituric acid), TC (total cholesterol), TDDP (transdermal double-layer patch), TG (triglycerides), VCAM-1 (vascular cell adhesion molecule 1), W/O (water-in-oil), WHO (World Health Organization), WT (wild-type), XO (xanthine oxidase).
